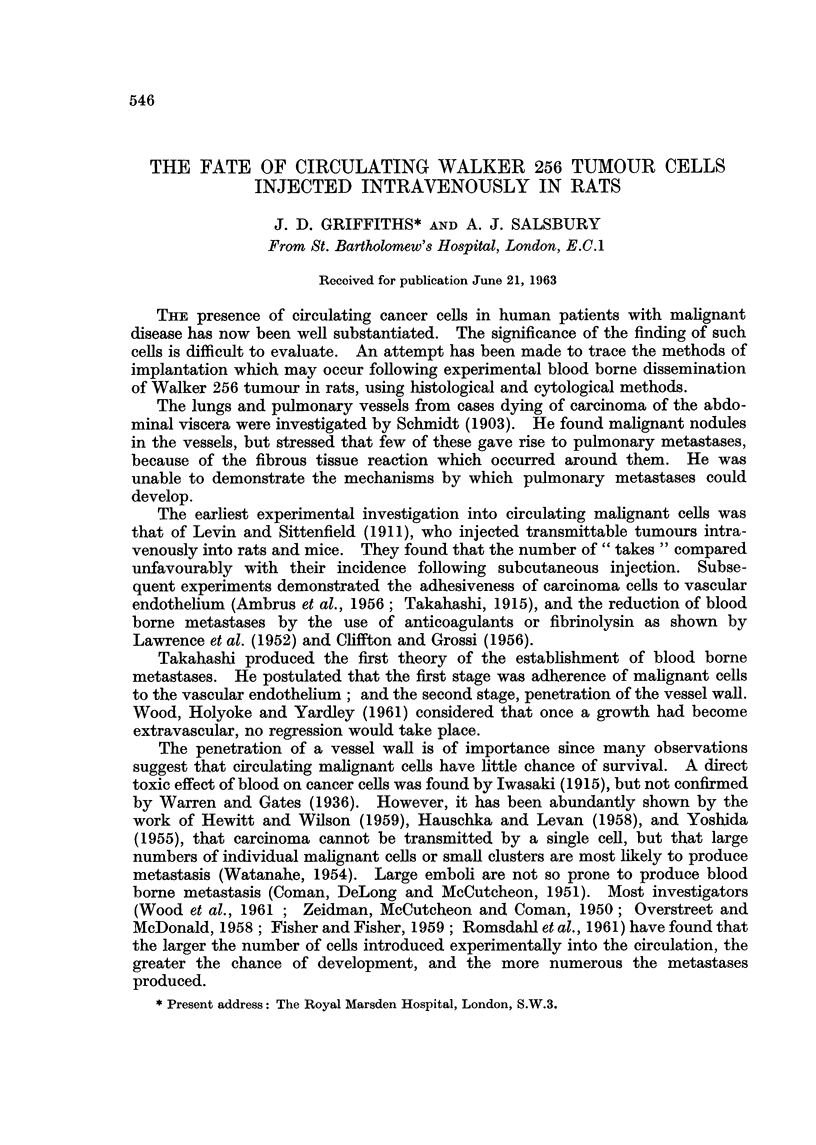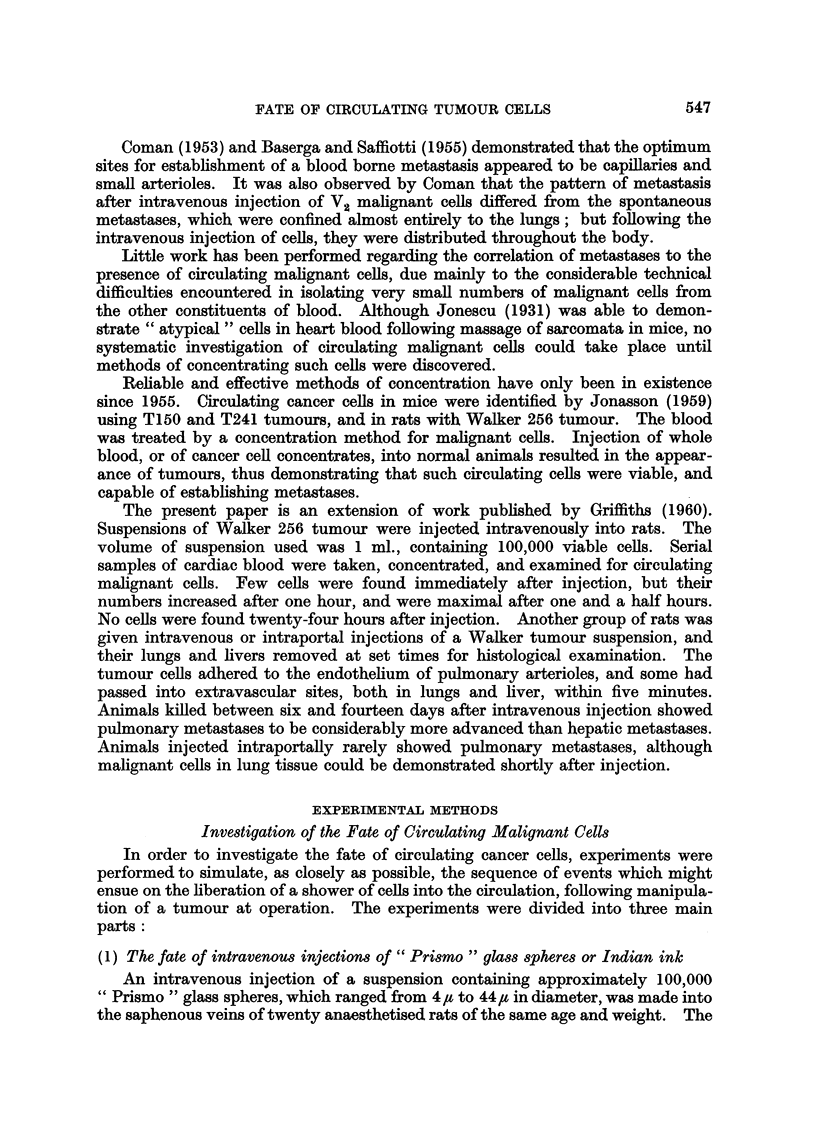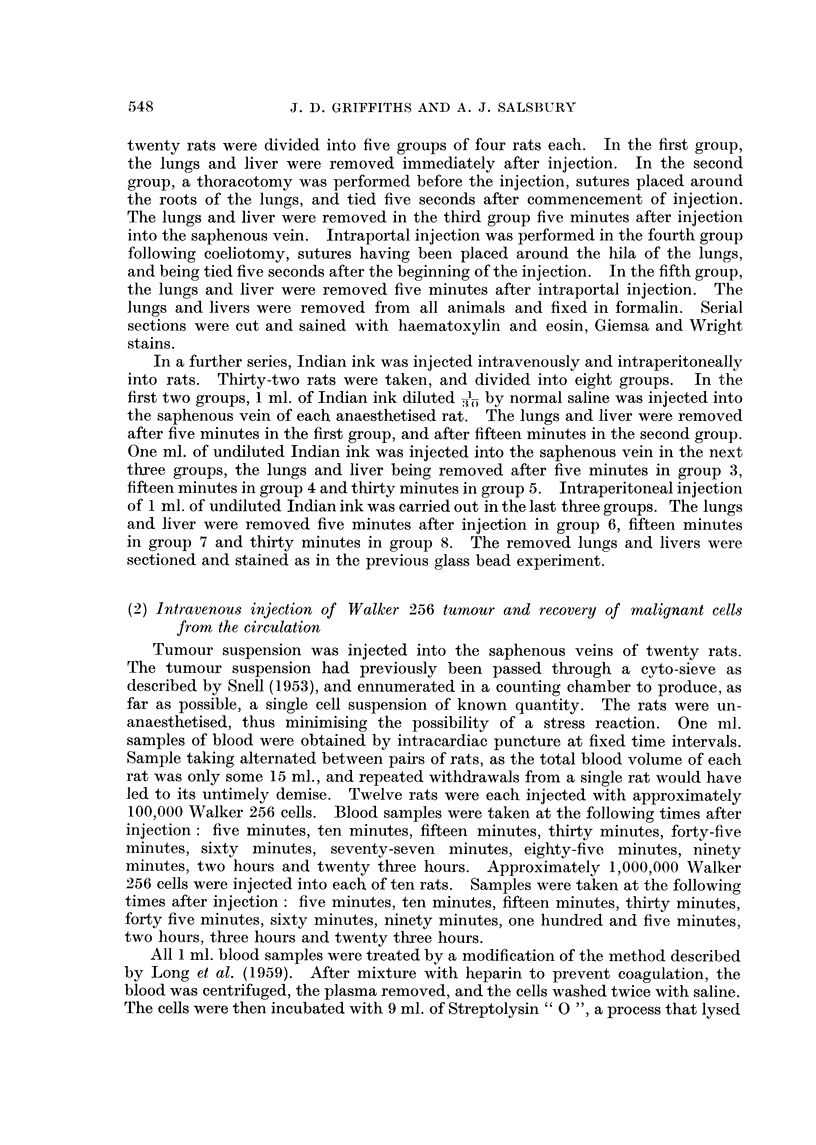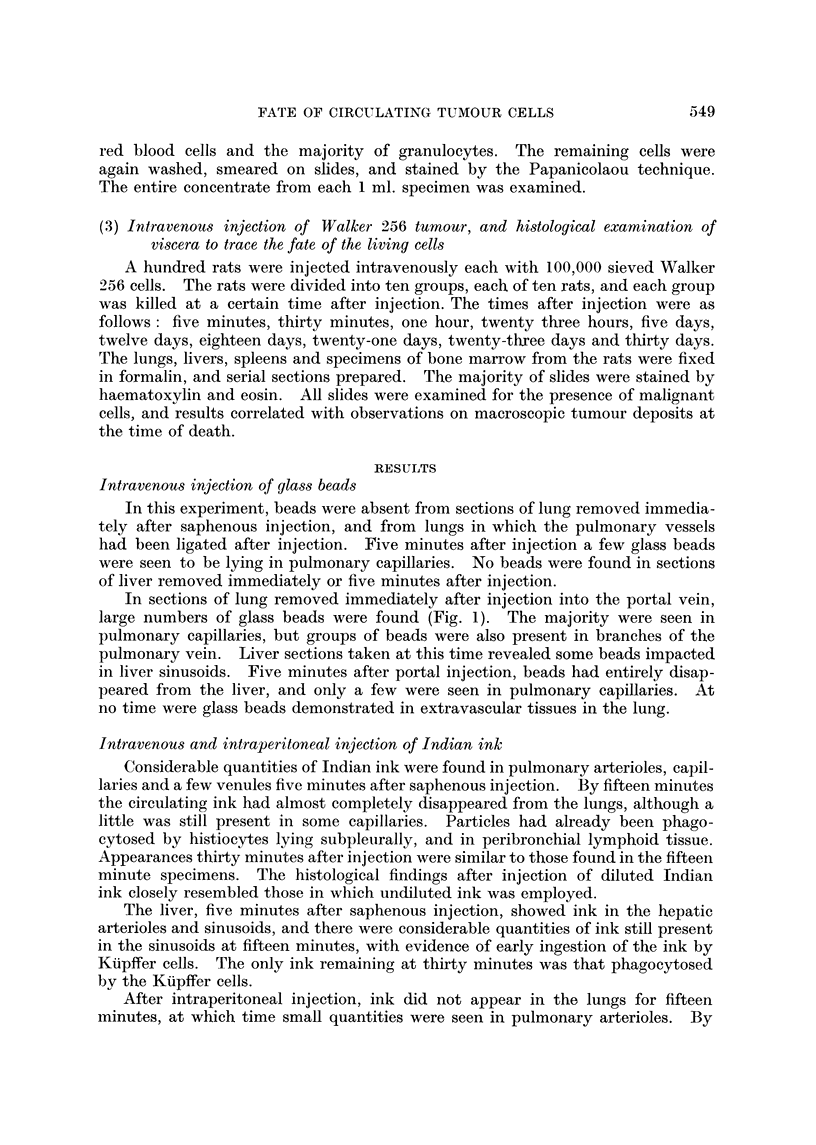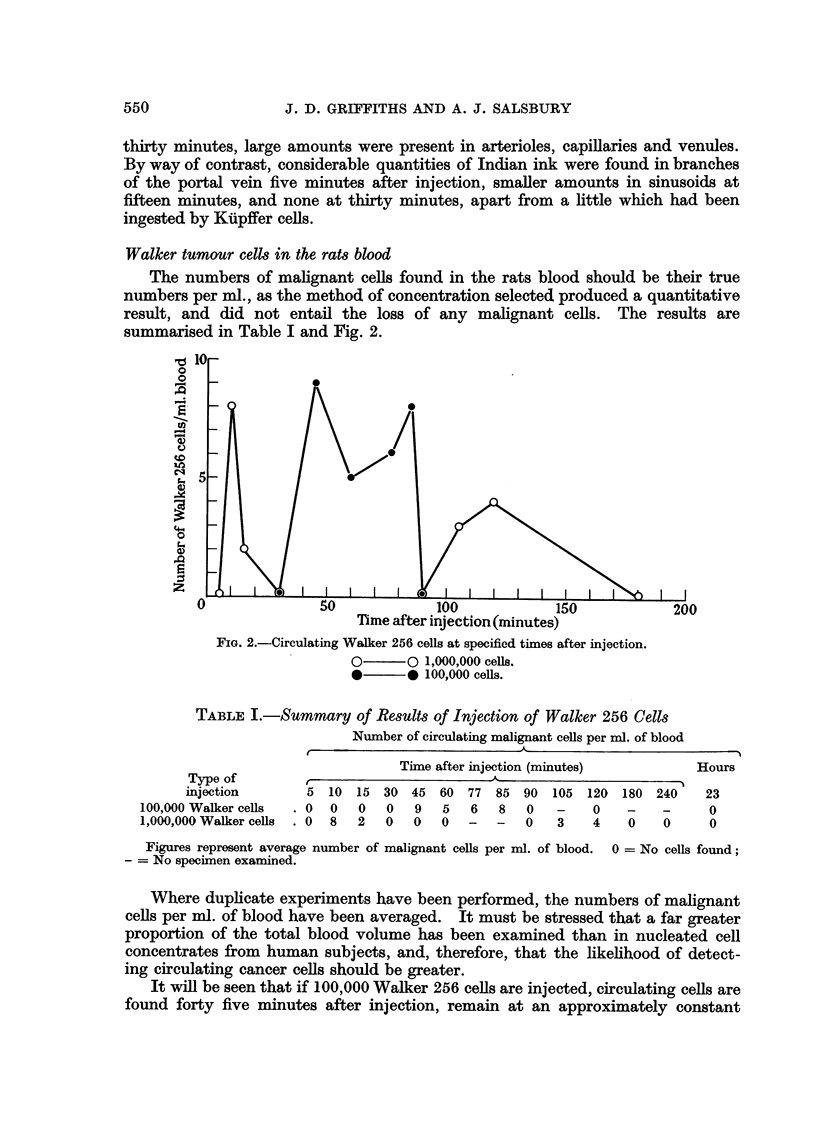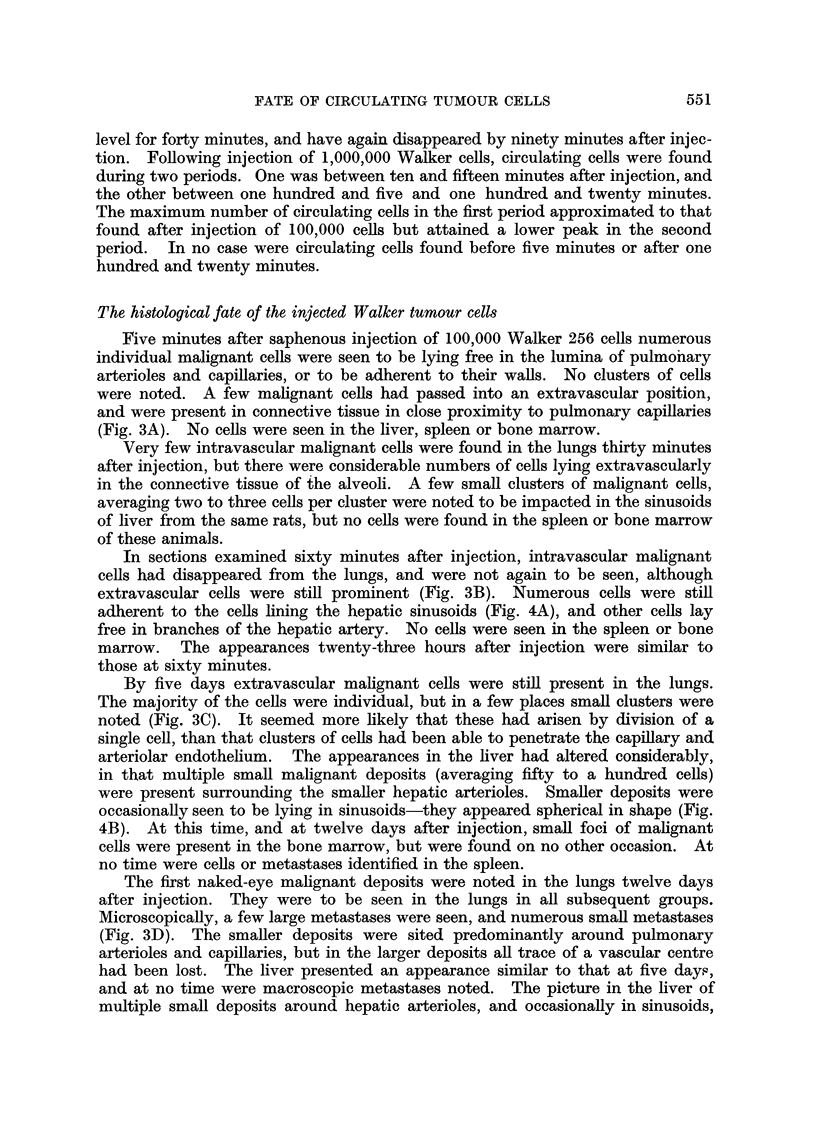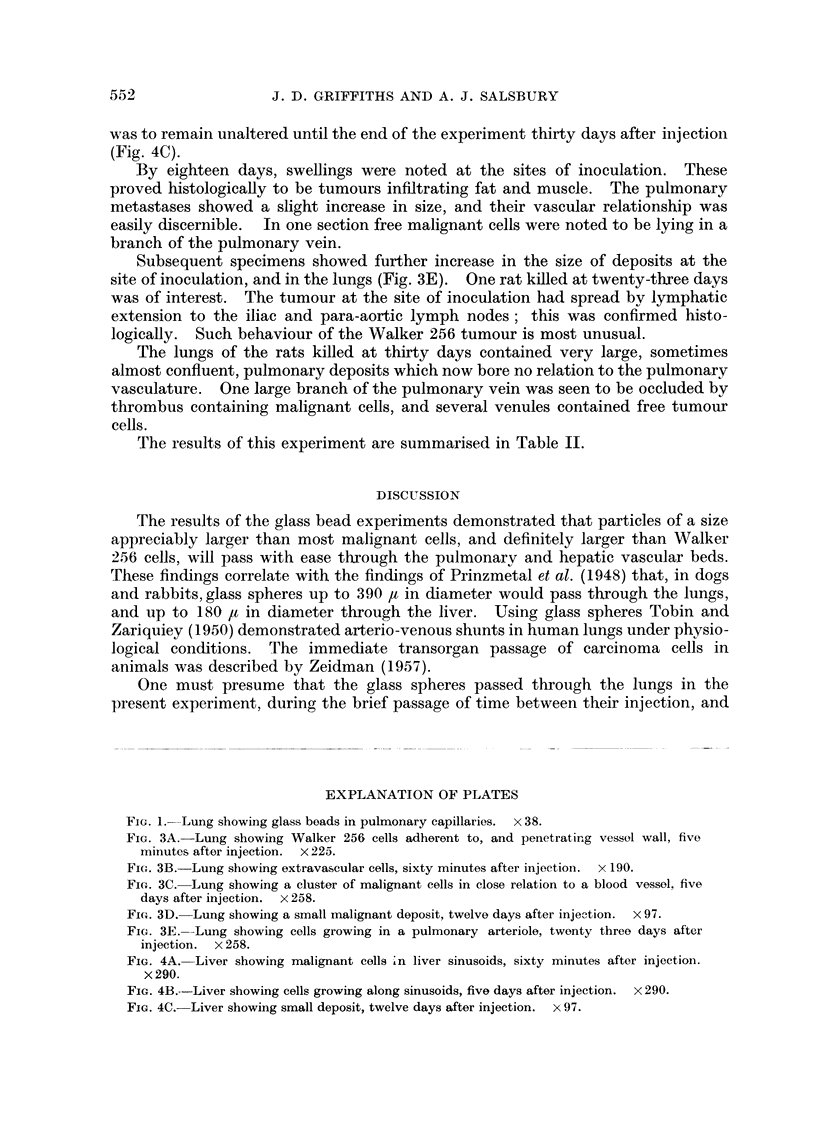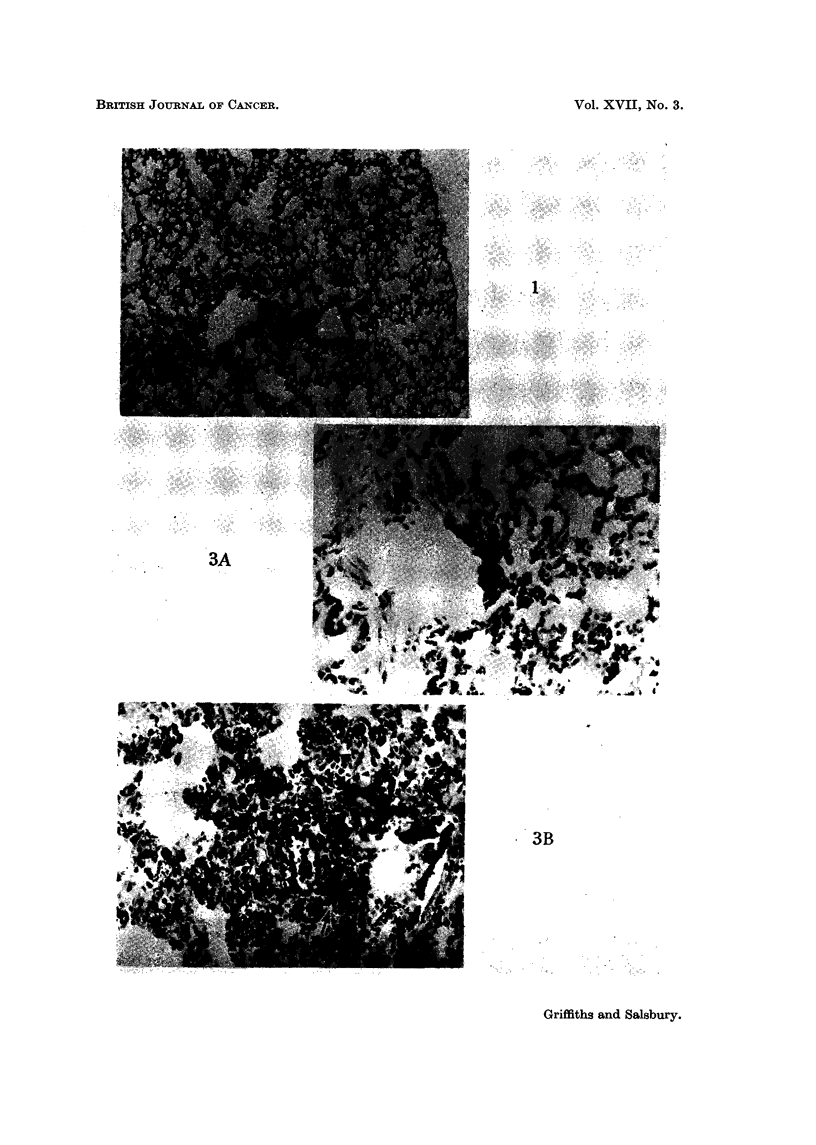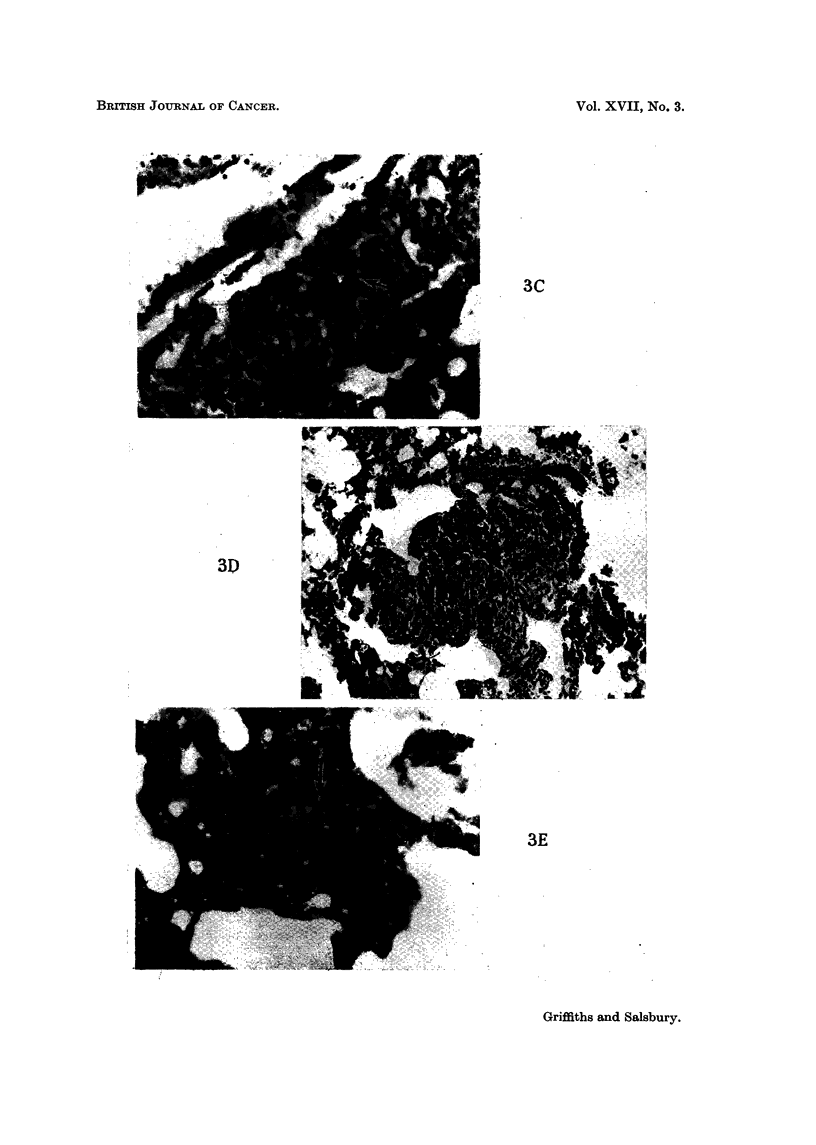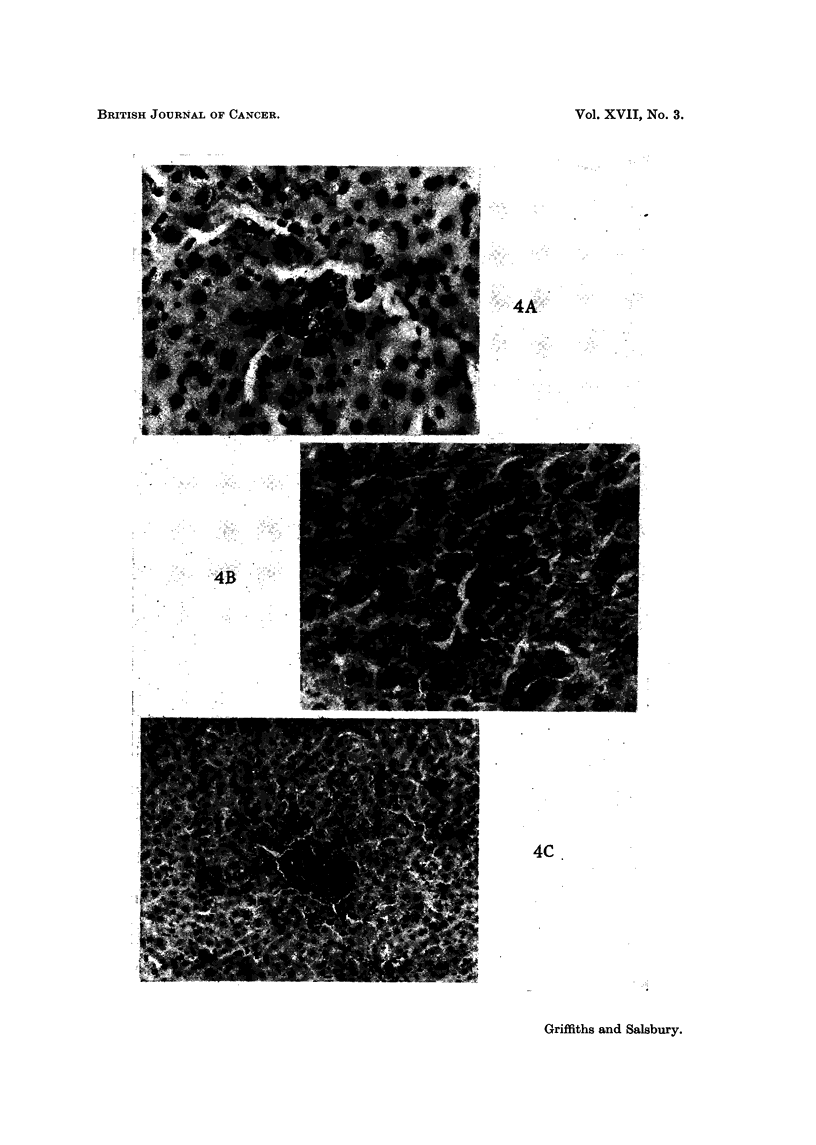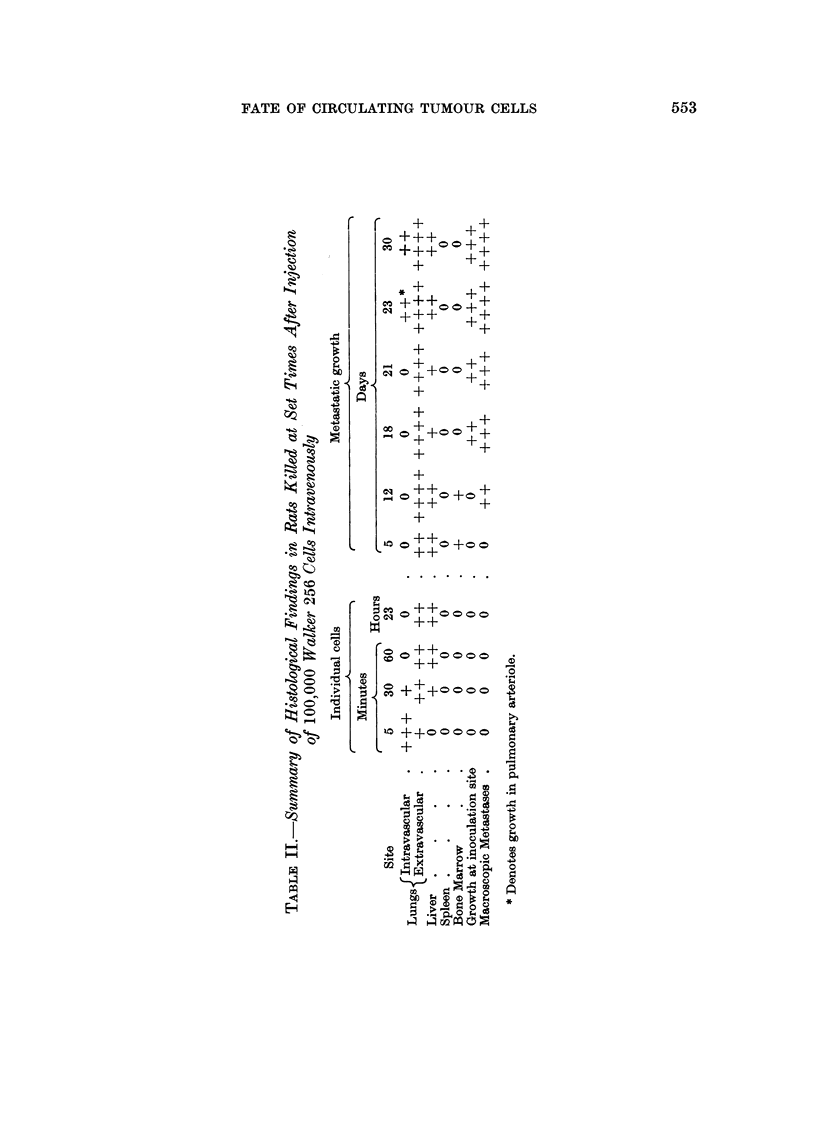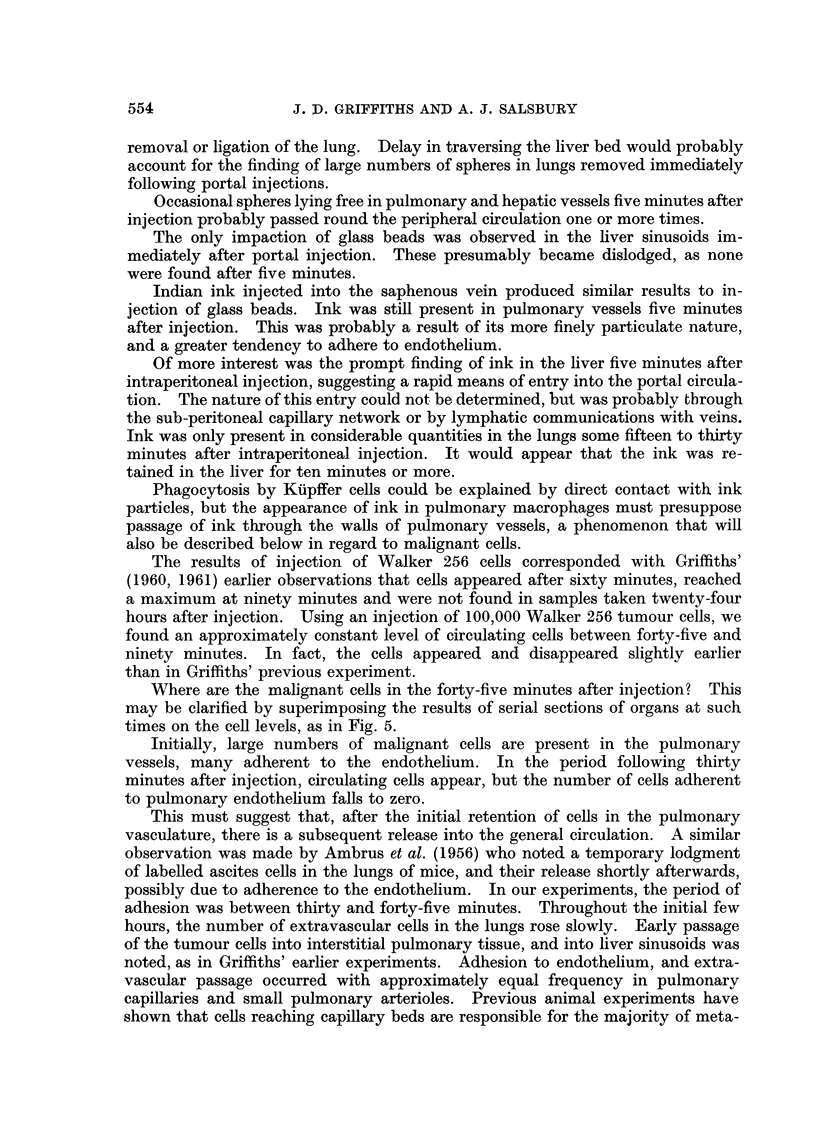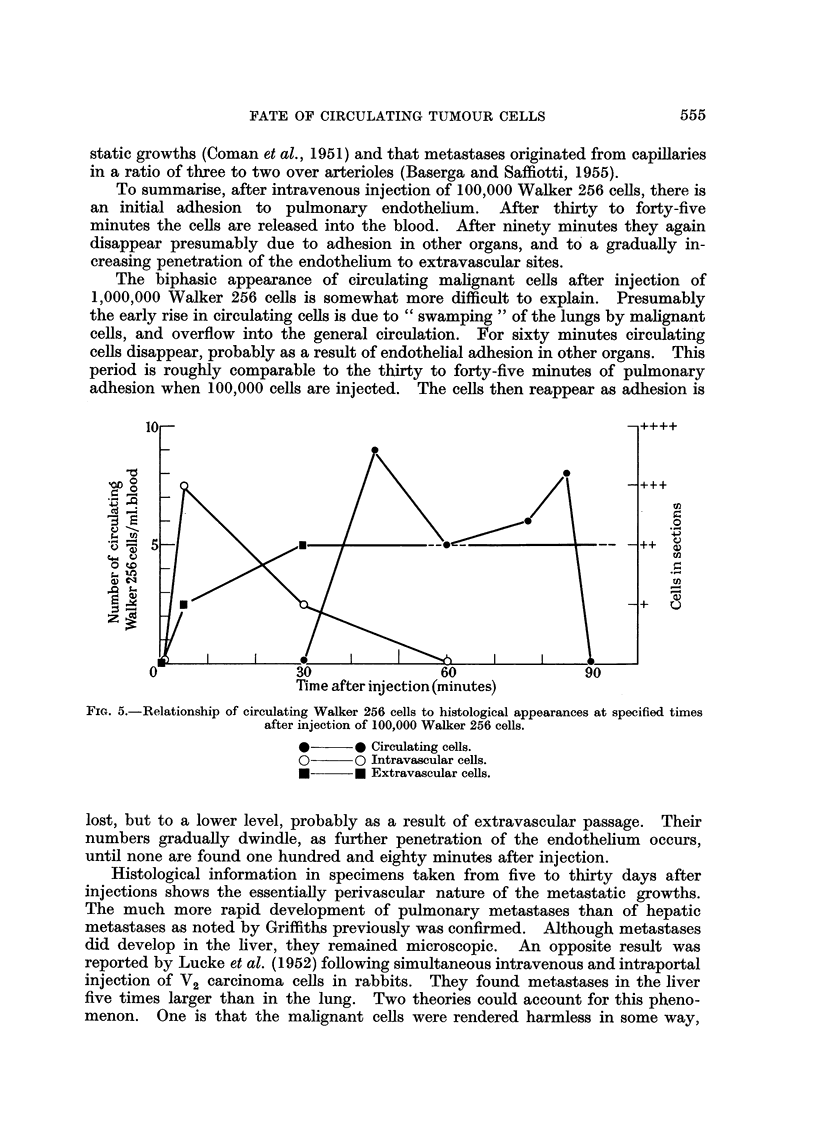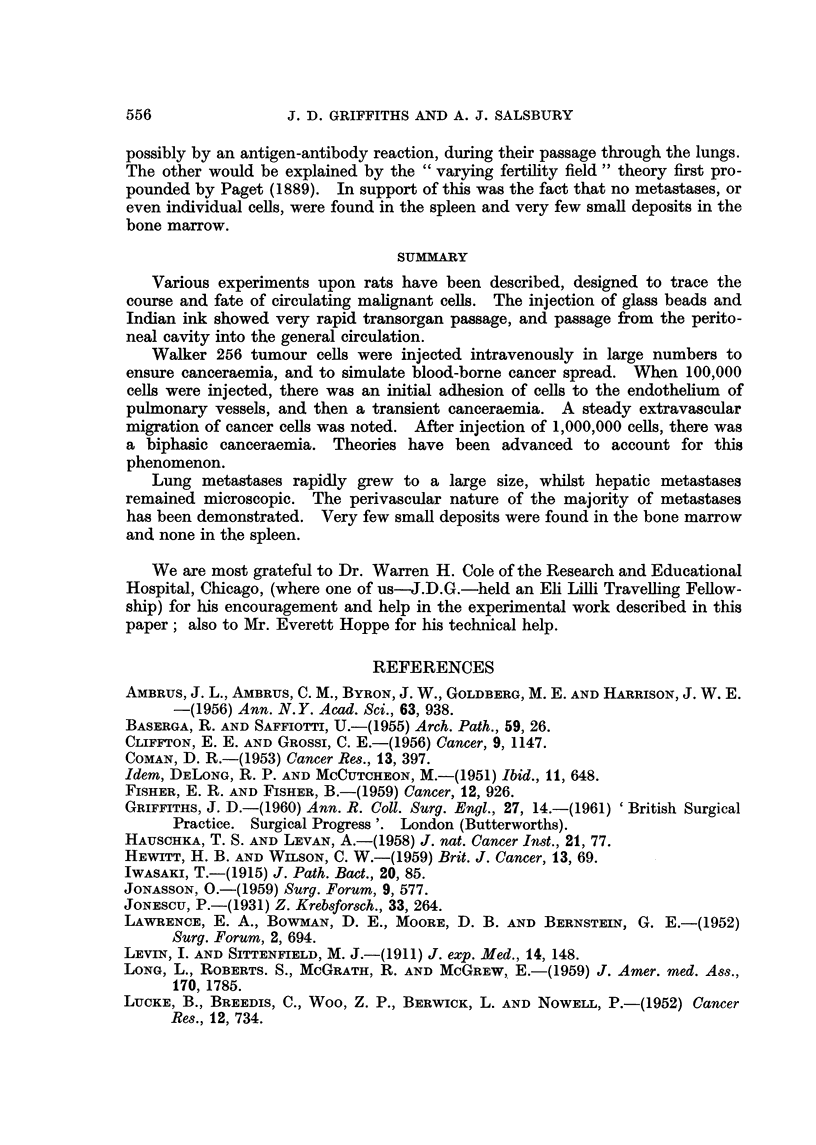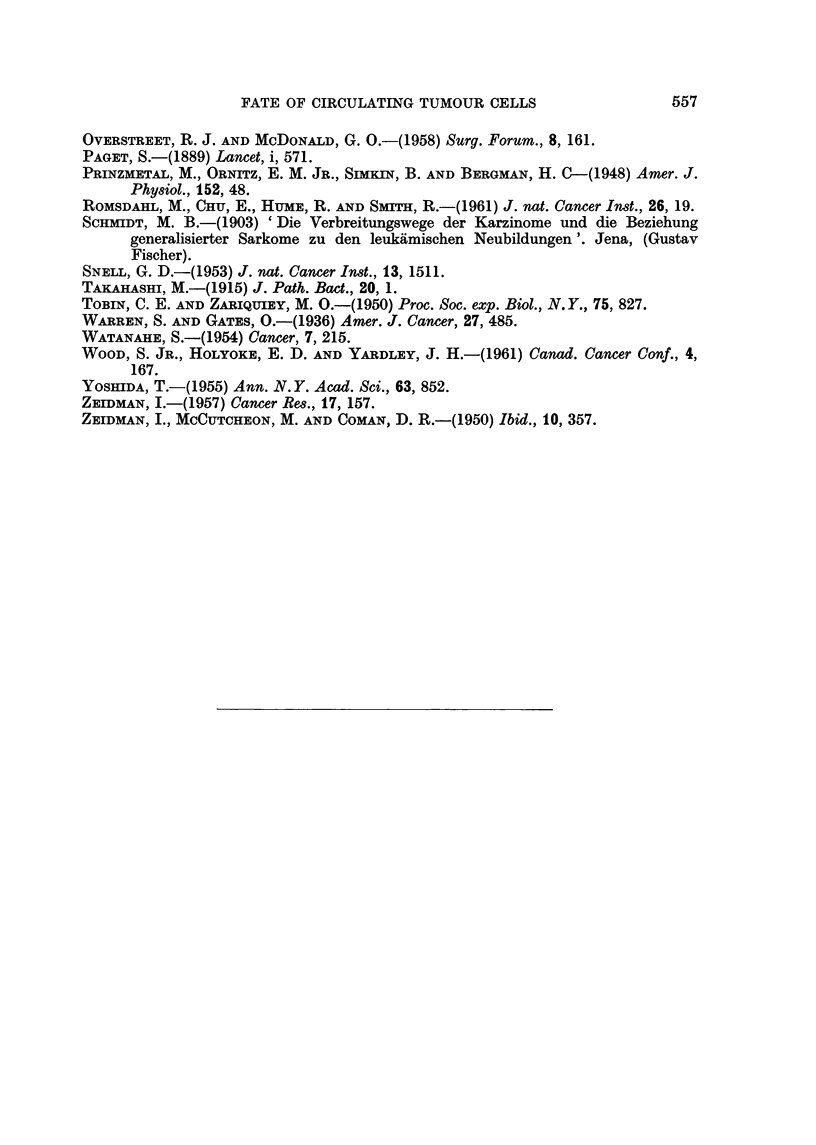# The Fate of Circulating Walker 256 Tumour Cells injected Intravenously in Rats

**DOI:** 10.1038/bjc.1963.73

**Published:** 1963-09

**Authors:** J. D. Griffiths, A. J. Salsbury

## Abstract

**Images:**


### THE FATE OF CIRCULATING WALKER 256 TUMOUR CELLS INJECTED INTRAVENOUSLY IN RATS

J. D. GRIFFITHS* AND A. J. SALSBURY
*From St. Bartholomew's Hospital, London, E.C.*1

Received for publication June 21, 1963

THE presence of circulating cancer cells in human patients with malignant disease has now been well substantiated. The significance of the finding of such cells is difficult to evaluate. An attempt has been made to trace the methods of implantation which may occur following experimental blood borne dissemination of Walker 256 tumour in rats, using histological and cytological methods.

The lungs and pulmonary vessels from cases dying of carcinoma of the abdominal viscera were investigated by Schmidt (1903). He found malignant nodules in the vessels, but stressed that few of these gave rise to pulmonary metastases, because of the fibrous tissue reaction which occurred around them. He was unable to demonstrate the mechanisms by which pulmonary metastases could develop.

The earliest experimental investigation into circulating malignant cells was that of Levin and Sittenfield (1911), who injected transmittable tumours intravenously into rats and mice. They found that the number of " takes " compared unfavourably with their incidence following subcutaneous injection. Subsequent experiments demonstrated the adhesiveness of carcinoma cells to vascular endothelium (Ambrus *et al.*, 1956 ; Takahashi, 1915), and the reduction of blood borne metastases by the use of anticoagulants or fibrinolysin as shown by Lawrence *et al.* (1952) and Cliffton and Grossi (1956).

Takahashi produced the first theory of the establishment of blood borne metastases. He postulated that the first stage was adherence of malignant cells to the vascular endothelium ; and the second stage, penetration of the vessel wall. Wood, Holyoke and Yardley (1961) considered that once a growth had become extravascular, no regression would take place.

The penetration of a vessel wall is of importance since many observations suggest that circulating malignant cells have little chance of survival. A direct toxic effect of blood on cancer cells was found by Iwasaki (1915), but not confirmed by Warren and Gates (1936). However, it has been abundantly shown by the work of Hewitt and Wilson (1959), Hauschka and Levan (1958), and Yoshida (1955), that carcinoma cannot be transmitted by a single cell, but that large numbers of individual malignant cells or small clusters are most likely to produce metastasis (Watanahe, 1954). Large emboli are not so prone to produce blood borne metastasis (Coman, DeLong and McCutcheon, 1951). Most investigators (Wood *et al.*, 1961 ; Zeidman, McCutcheon and Coman, 1950 ; Overstreet and McDonald, 1958 ; Fisher and Fisher, 1959 ; Romsdahl *et al.*, 1961) have found that the larger the number of cells introduced experimentally into the circulation, the greater the chance of development, and the more numerous the metastases produced.

* Present address: The Royal Marsden Hospital, London, S.W.3.

Coman (1953) and Baserga and Saffiotti (1955) demonstrated that the optimum sites for establishment of a blood borne metastasis appeared to be capillaries and small arterioles. It was also observed by Coman that the pattern of metastasis after intravenous injection of $V_2$ malignant cells differed from the spontaneous metastases, which were confined almost entirely to the lungs ; but following the intravenous injection of cells, they were distributed throughout the body.

Little work has been performed regarding the correlation of metastases to the presence of circulating malignant cells, due mainly to the considerable technical difficulties encountered in isolating very small numbers of malignant cells from the other constituents of blood. Although Jonescu (1931) was able to demonstrate " atypical " cells in heart blood following massage of sarcomata in mice, no systematic investigation of circulating malignant cells could take place until methods of concentrating such cells were discovered.

Reliable and effective methods of concentration have only been in existence since 1955. Circulating cancer cells in mice were identified by Jonasson (1959) using T150 and T241 tumours, and in rats with Walker 256 tumour. The blood was treated by a concentration method for malignant cells. Injection of whole blood, or of cancer cell concentrates, into normal animals resulted in the appearance of tumours, thus demonstrating that such circulating cells were viable, and capable of establishing metastases.

The present paper is an extension of work published by Griffiths (1960). Suspensions of Walker 256 tumour were injected intravenously into rats. The volume of suspension used was 1 ml., containing 100,000 viable cells. Serial samples of cardiac blood were taken, concentrated, and examined for circulating malignant cells. Few cells were found immediately after injection, but their numbers increased after one hour, and were maximal after one and a half hours. No cells were found twenty-four hours after injection. Another group of rats was given intravenous or intraportal injections of a Walker tumour suspension, and their lungs and livers removed at set times for histological examination. The tumour cells adhered to the endothelium of pulmonary arterioles, and some had passed into extravascular sites, both in lungs and liver, within five minutes. Animals killed between six and fourteen days after intravenous injection showed pulmonary metastases to be considerably more advanced than hepatic metastases. Animals injected intraportally rarely showed pulmonary metastases, although malignant cells in lung tissue could be demonstrated shortly after injection.

### EXPERIMENTAL METHODS

### *Investigation of the Fate of Circulating Malignant Cells*

In order to investigate the fate of circulating cancer cells, experiments were performed to simulate, as closely as possible, the sequence of events which might ensue on the liberation of a shower of cells into the circulation, following manipulation of a tumour at operation. The experiments were divided into three main parts :

(1) *The fate of intravenous injections of " Prismo " glass spheres or Indian ink*

An intravenous injection of a suspension containing approximately 100,000 " Prismo " glass spheres, which ranged from $4\mu$ to $44\mu$ in diameter, was made into the saphenous veins of twenty anaesthetised rats of the same age and weight. The twenty rats were divided into five groups of four rats each. In the first group, the lungs and liver were removed immediately after injection. In the second group, a thoracotomy was performed before the injection, sutures placed around the roots of the lungs, and tied five seconds after commencement of injection. The lungs and liver were removed in the third group five minutes after injection into the saphenous vein. Intraportal injection was performed in the fourth group following coeliotomy, sutures having been placed around the hila of the lungs, and being tied five seconds after the beginning of the injection. In the fifth group, the lungs and liver were removed five minutes after intraportal injection. The lungs and livers were removed from all animals and fixed in formalin. Serial sections were cut and sained with haematoxylin and eosin, Giemsa and Wright stains.

In a further series, Indian ink was injected intravenously and intraperitoneally into rats. Thirty-two rats were taken, and divided into eight groups. In the first two groups, 1 ml. of Indian ink diluted $\frac{1}{30}$ by normal saline was injected into the saphenous vein of each anaesthetised rat. The lungs and liver were removed after five minutes in the first group, and after fifteen minutes in the second group. One ml. of undiluted Indian ink was injected into the saphenous vein in the next three groups, the lungs and liver being removed after five minutes in group 3, fifteen minutes in group 4 and thirty minutes in group 5. Intraperitoneal injection of 1 ml. of undiluted Indian ink was carried out in the last three groups. The lungs and liver were removed five minutes after injection in group 6, fifteen minutes in group 7 and thirty minutes in group 8. The removed lungs and livers were sectioned and stained as in the previous glass bead experiment.

(2) *Intravenous injection of Walker 256 tumour and recovery of malignant cells from the circulation*

Tumour suspension was injected into the saphenous veins of twenty rats. The tumour suspension had previously been passed through a cyto-sieve as described by Snell (1953), and ennumerated in a counting chamber to produce, as far as possible, a single cell suspension of known quantity. The rats were unanaesthetised, thus minimising the possibility of a stress reaction. One ml. samples of blood were obtained by intracardiac puncture at fixed time intervals. Sample taking alternated between pairs of rats, as the total blood volume of each rat was only some 15 ml., and repeated withdrawals from a single rat would have led to its untimely demise. Twelve rats were each injected with approximately 100,000 Walker 256 cells. Blood samples were taken at the following times after injection : five minutes, ten minutes, fifteen minutes, thirty minutes, forty-five minutes, sixty minutes, seventy-seven minutes, eighty-five minutes, ninety minutes, two hours and twenty three hours. Approximately 1,000,000 Walker 256 cells were injected into each of ten rats. Samples were taken at the following times after injection : five minutes, ten minutes, fifteen minutes, thirty minutes, forty five minutes, sixty minutes, ninety minutes, one hundred and five minutes, two hours, three hours and twenty three hours.

All 1 ml. blood samples were treated by a modification of the method described by Long *et al.* (1959). After mixture with heparin to prevent coagulation, the blood was centrifuged, the plasma removed, and the cells washed twice with saline. The cells were then incubated with 9 ml. of Streptolysin " O ", a process that lysed red blood cells and the majority of granulocytes. The remaining cells were again washed, smeared on slides, and stained by the Papanicolaou technique. The entire concentrate from each 1 ml. specimen was examined.

(3) *Intravenous injection of Walker 256 tumour, and histological examination of viscera to trace the fate of the living cells*

A hundred rats were injected intravenously each with 100,000 sieved Walker 256 cells. The rats were divided into ten groups, each of ten rats, and each group was killed at a certain time after injection. The times after injection were as follows : five minutes, thirty minutes, one hour, twenty three hours, five days, twelve days, eighteen days, twenty-one days, twenty-three days and thirty days. The lungs, livers, spleens and specimens of bone marrow from the rats were fixed in formalin, and serial sections prepared. The majority of slides were stained by haematoxylin and eosin. All slides were examined for the presence of malignant cells, and results correlated with observations on macroscopic tumour deposits at the time of death.

### RESULTS

*Intravenous injection of glass beads*

In this experiment, beads were absent from sections of lung removed immediately after saphenous injection, and from lungs in which the pulmonary vessels had been ligated after injection. Five minutes after injection a few glass beads were seen to be lying in pulmonary capillaries. No beads were found in sections of liver removed immediately or five minutes after injection.

In sections of lung removed immediately after injection into the portal vein, large numbers of glass beads were found (Fig. 1). The majority were seen in pulmonary capillaries, but groups of beads were also present in branches of the pulmonary vein. Liver sections taken at this time revealed some beads impacted in liver sinusoids. Five minutes after portal injection, beads had entirely disappeared from the liver, and only a few were seen in pulmonary capillaries. At no time were glass beads demonstrated in extravascular tissues in the lung.

*Intravenous and intraperitoneal injection of Indian ink*

Considerable quantities of Indian ink were found in pulmonary arterioles, capillaries and a few venules five minutes after saphenous injection. By fifteen minutes the circulating ink had almost completely disappeared from the lungs, although a little was still present in some capillaries. Particles had already been phagocytosed by histiocytes lying subpleurally, and in peribronchial lymphoid tissue. Appearances thirty minutes after injection were similar to those found in the fifteen minute specimens. The histological findings after injection of diluted Indian ink closely resembled those in which undiluted ink was employed.

The liver, five minutes after saphenous injection, showed ink in the hepatic arterioles and sinusoids, and there were considerable quantities of ink still present in the sinusoids at fifteen minutes, with evidence of early ingestion of the ink by Küpffer cells. The only ink remaining at thirty minutes was that phagocytosed by the Küpffer cells.

After intraperitoneal injection, ink did not appear in the lungs for fifteen minutes, at which time small quantities were seen in pulmonary arterioles. By thirty minutes, large amounts were present in arterioles, capillaries and venules. By way of contrast, considerable quantities of Indian ink were found in branches of the portal vein five minutes after injection, smaller amounts in sinusoids at fifteen minutes, and none at thirty minutes, apart from a little which had been ingested by Küpffer cells.

*Walker tumour cells in the rats blood*

The numbers of malignant cells found in the rats blood should be their true numbers per ml., as the method of concentration selected produced a quantitative result, and did not entail the loss of any malignant cells. The results are summarised in Table I and Fig. 2.

FIG. 2.—Circulating Walker 256 cells at specified times after injection.
○———○ 1,000,000 cells.
●———● 100,000 cells.

TABLE I.—*Summary of Results of Injection of Walker 256 Cells*

| Type of injection | Number of circulating malignant cells per ml. of blood: Time after injection (minutes) 5 | 10 | 15 | 30 | 45 | 60 | 77 | 85 | 90 | 105 | 120 | 180 | 240 | Hours 23 |
|---|---|---|---|---|---|---|---|---|---|---|---|---|---|---|
| 100,000 Walker cells . | 0 | 0 | 0 | 0 | 9 | 5 | 6 | 8 | 0 | – | 0 | – | – | 0 |
| 1,000,000 Walker cells . | 0 | 8 | 2 | 0 | 0 | 0 | – | – | 0 | 3 | 4 | 0 | 0 | 0 |

Figures represent average number of malignant cells per ml. of blood. 0 = No cells found; – = No specimen examined.

Where duplicate experiments have been performed, the numbers of malignant cells per ml. of blood have been averaged. It must be stressed that a far greater proportion of the total blood volume has been examined than in nucleated cell concentrates from human subjects, and, therefore, that the likelihood of detecting circulating cancer cells should be greater.

It will be seen that if 100,000 Walker 256 cells are injected, circulating cells are found forty five minutes after injection, remain at an approximately constant level for forty minutes, and have again disappeared by ninety minutes after injection. Following injection of 1,000,000 Walker cells, circulating cells were found during two periods. One was between ten and fifteen minutes after injection, and the other between one hundred and five and one hundred and twenty minutes. The maximum number of circulating cells in the first period approximated to that found after injection of 100,000 cells but attained a lower peak in the second period. In no case were circulating cells found before five minutes or after one hundred and twenty minutes.

*The histological fate of the injected Walker tumour cells*

Five minutes after saphenous injection of 100,000 Walker 256 cells numerous individual malignant cells were seen to be lying free in the lumina of pulmonary arterioles and capillaries, or to be adherent to their walls. No clusters of cells were noted. A few malignant cells had passed into an extravascular position, and were present in connective tissue in close proximity to pulmonary capillaries (Fig. 3A). No cells were seen in the liver, spleen or bone marrow.

Very few intravascular malignant cells were found in the lungs thirty minutes after injection, but there were considerable numbers of cells lying extravascularly in the connective tissue of the alveoli. A few small clusters of malignant cells, averaging two to three cells per cluster were noted to be impacted in the sinusoids of liver from the same rats, but no cells were found in the spleen or bone marrow of these animals.

In sections examined sixty minutes after injection, intravascular malignant cells had disappeared from the lungs, and were not again to be seen, although extravascular cells were still prominent (Fig. 3B). Numerous cells were still adherent to the cells lining the hepatic sinusoids (Fig. 4A), and other cells lay free in branches of the hepatic artery. No cells were seen in the spleen or bone marrow. The appearances twenty-three hours after injection were similar to those at sixty minutes.

By five days extravascular malignant cells were still present in the lungs. The majority of the cells were individual, but in a few places small clusters were noted (Fig. 3C). It seemed more likely that these had arisen by division of a single cell, than that clusters of cells had been able to penetrate the capillary and arteriolar endothelium. The appearances in the liver had altered considerably, in that multiple small malignant deposits (averaging fifty to a hundred cells) were present surrounding the smaller hepatic arterioles. Smaller deposits were occasionally seen to be lying in sinusoids—they appeared spherical in shape (Fig. 4B). At this time, and at twelve days after injection, small foci of malignant cells were present in the bone marrow, but were found on no other occasion. At no time were cells or metastases identified in the spleen.

The first naked-eye malignant deposits were noted in the lungs twelve days after injection. They were to be seen in the lungs in all subsequent groups. Microscopically, a few large metastases were seen, and numerous small metastases (Fig. 3D). The smaller deposits were sited predominantly around pulmonary arterioles and capillaries, but in the larger deposits all trace of a vascular centre had been lost. The liver presented an appearance similar to that at five days, and at no time were macroscopic metastases noted. The picture in the liver of multiple small deposits around hepatic arterioles, and occasionally in sinusoids, was to remain unaltered until the end of the experiment thirty days after injection (Fig. 4C).

By eighteen days, swellings were noted at the sites of inoculation. These proved histologically to be tumours infiltrating fat and muscle. The pulmonary metastases showed a slight increase in size, and their vascular relationship was easily discernible. In one section free malignant cells were noted to be lying in a branch of the pulmonary vein.

Subsequent specimens showed further increase in the size of deposits at the site of inoculation, and in the lungs (Fig. 3E). One rat killed at twenty-three days was of interest. The tumour at the site of inoculation had spread by lymphatic extension to the iliac and para-aortic lymph nodes ; this was confirmed histologically. Such behaviour of the Walker 256 tumour is most unusual.

The lungs of the rats killed at thirty days contained very large, sometimes almost confluent, pulmonary deposits which now bore no relation to the pulmonary vasculature. One large branch of the pulmonary vein was seen to be occluded by thrombus containing malignant cells, and several venules contained free tumour cells.

The results of this experiment are summarised in Table II.

### DISCUSSION

The results of the glass bead experiments demonstrated that particles of a size appreciably larger than most malignant cells, and definitely larger than Walker 256 cells, will pass with ease through the pulmonary and hepatic vascular beds. These findings correlate with the findings of Prinzmetal *et al.* (1948) that, in dogs and rabbits, glass spheres up to 390 $\mu$ in diameter would pass through the lungs, and up to 180 $\mu$ in diameter through the liver. Using glass spheres Tobin and Zariquiey (1950) demonstrated arterio-venous shunts in human lungs under physiological conditions. The immediate transorgan passage of carcinoma cells in animals was described by Zeidman (1957).

One must presume that the glass spheres passed through the lungs in the present experiment, during the brief passage of time between their injection, and

---

### EXPLANATION OF PLATES

FIG. 1.—Lung showing glass beads in pulmonary capillaries. ×38.

FIG. 3A.—Lung showing Walker 256 cells adherent to, and penetrating vessel wall, five minutes after injection. ×225.

FIG. 3B.—Lung showing extravascular cells, sixty minutes after injection. ×190.

FIG. 3C.—Lung showing a cluster of malignant cells in close relation to a blood vessel, five days after injection. ×258.

FIG. 3D.—Lung showing a small malignant deposit, twelve days after injection. ×97.

FIG. 3E.—Lung showing cells growing in a pulmonary arteriole, twenty three days after injection. ×258.

FIG. 4A.—Liver showing malignant cells in liver sinusoids, sixty minutes after injection. ×290.

FIG. 4B.—Liver showing cells growing along sinusoids, five days after injection. ×290.

FIG. 4C.—Liver showing small deposit, twelve days after injection. ×97.

1

3A

3B

3C

3D

3E

Griffiths and Salsbury.

4A

4B

4C

TABLE II.—*Summary of Histological Findings in Rats Killed at Set Times After Injection of* 100,000 *Walker* 256 *Cells Intravenously*

| Site | Individual cells: Minutes 5 | 30 | 60 | Hours 23 | Metastatic growth: Days 5 | 12 | 18 | 21 | 23 | 30 |
|---|---|---|---|---|---|---|---|---|---|---|
| Lungs Intravascular | +++ | + | 0 | 0 | 0 | 0 | 0 | 0 | ++* | ++ |
| Lungs Extravascular | + | ++ | ++ | ++ | ++ | ++++ | ++++ | ++++ | ++++ | ++++ |
| Liver | 0 | + | ++ | ++ | ++ | ++ | + | + | ++ | ++ |
| Spleen | 0 | 0 | 0 | 0 | 0 | 0 | 0 | 0 | 0 | 0 |
| Bone Marrow | 0 | 0 | 0 | 0 | + | + | 0 | 0 | 0 | 0 |
| Growth at inoculation site | 0 | 0 | 0 | 0 | 0 | 0 | ++ | ++ | +++ | +++ |
| Macroscopic Metastases | 0 | 0 | 0 | 0 | 0 | ++ | +++ | +++ | ++++ | ++++ |

* Denotes growth in pulmonary arteriole.

removal or ligation of the lung. Delay in traversing the liver bed would probably account for the finding of large numbers of spheres in lungs removed immediately following portal injections.

Occasional spheres lying free in pulmonary and hepatic vessels five minutes after injection probably passed round the peripheral circulation one or more times.

The only impaction of glass beads was observed in the liver sinusoids immediately after portal injection. These presumably became dislodged, as none were found after five minutes.

Indian ink injected into the saphenous vein produced similar results to injection of glass beads. Ink was still present in pulmonary vessels five minutes after injection. This was probably a result of its more finely particulate nature, and a greater tendency to adhere to endothelium.

Of more interest was the prompt finding of ink in the liver five minutes after intraperitoneal injection, suggesting a rapid means of entry into the portal circulation. The nature of this entry could not be determined, but was probably through the sub-peritoneal capillary network or by lymphatic communications with veins. Ink was only present in considerable quantities in the lungs some fifteen to thirty minutes after intraperitoneal injection. It would appear that the ink was retained in the liver for ten minutes or more.

Phagocytosis by Küpffer cells could be explained by direct contact with ink particles, but the appearance of ink in pulmonary macrophages must presuppose passage of ink through the walls of pulmonary vessels, a phenomenon that will also be described below in regard to malignant cells.

The results of injection of Walker 256 cells corresponded with Griffiths' (1960, 1961) earlier observations that cells appeared after sixty minutes, reached a maximum at ninety minutes and were not found in samples taken twenty-four hours after injection. Using an injection of 100,000 Walker 256 tumour cells, we found an approximately constant level of circulating cells between forty-five and ninety minutes. In fact, the cells appeared and disappeared slightly earlier than in Griffiths' previous experiment.

Where are the malignant cells in the forty-five minutes after injection? This may be clarified by superimposing the results of serial sections of organs at such times on the cell levels, as in Fig. 5.

Initially, large numbers of malignant cells are present in the pulmonary vessels, many adherent to the endothelium. In the period following thirty minutes after injection, circulating cells appear, but the number of cells adherent to pulmonary endothelium falls to zero.

This must suggest that, after the initial retention of cells in the pulmonary vasculature, there is a subsequent release into the general circulation. A similar observation was made by Ambrus *et al.* (1956) who noted a temporary lodgment of labelled ascites cells in the lungs of mice, and their release shortly afterwards, possibly due to adherence to the endothelium. In our experiments, the period of adhesion was between thirty and forty-five minutes. Throughout the initial few hours, the number of extravascular cells in the lungs rose slowly. Early passage of the tumour cells into interstitial pulmonary tissue, and into liver sinusoids was noted, as in Griffiths' earlier experiments. Adhesion to endothelium, and extravascular passage occurred with approximately equal frequency in pulmonary capillaries and small pulmonary arterioles. Previous animal experiments have shown that cells reaching capillary beds are responsible for the majority of metastatic growths (Coman *et al.*, 1951) and that metastases originated from capillaries in a ratio of three to two over arterioles (Baserga and Saffiotti, 1955).

To summarise, after intravenous injection of 100,000 Walker 256 cells, there is an initial adhesion to pulmonary endothelium. After thirty to forty-five minutes the cells are released into the blood. After ninety minutes they again disappear presumably due to adhesion in other organs, and to a gradually increasing penetration of the endothelium to extravascular sites.

The biphasic appearance of circulating malignant cells after injection of 1,000,000 Walker 256 cells is somewhat more difficult to explain. Presumably the early rise in circulating cells is due to " swamping " of the lungs by malignant cells, and overflow into the general circulation. For sixty minutes circulating cells disappear, probably as a result of endothelial adhesion in other organs. This period is roughly comparable to the thirty to forty-five minutes of pulmonary adhesion when 100,000 cells are injected. The cells then reappear as adhesion is

FIG. 5.—Relationship of circulating Walker 256 cells to histological appearances at specified times after injection of 100,000 Walker 256 cells.

●———● Circulating cells.

○———○ Intravascular cells.

■———■ Extravascular cells.

lost, but to a lower level, probably as a result of extravascular passage. Their numbers gradually dwindle, as further penetration of the endothelium occurs, until none are found one hundred and eighty minutes after injection.

Histological information in specimens taken from five to thirty days after injections shows the essentially perivascular nature of the metastatic growths. The much more rapid development of pulmonary metastases than of hepatic metastases as noted by Griffiths previously was confirmed. Although metastases did develop in the liver, they remained microscopic. An opposite result was reported by Lucke *et al.* (1952) following simultaneous intravenous and intraportal injection of $V_2$ carcinoma cells in rabbits. They found metastases in the liver five times larger than in the lung. Two theories could account for this phenomenon. One is that the malignant cells were rendered harmless in some way, possibly by an antigen-antibody reaction, during their passage through the lungs. The other would be explained by the " varying fertility field " theory first propounded by Paget (1889). In support of this was the fact that no metastases, or even individual cells, were found in the spleen and very few small deposits in the bone marrow.

SUMMARY

Various experiments upon rats have been described, designed to trace the course and fate of circulating malignant cells. The injection of glass beads and Indian ink showed very rapid transorgan passage, and passage from the peritoneal cavity into the general circulation.

Walker 256 tumour cells were injected intravenously in large numbers to ensure canceraemia, and to simulate blood-borne cancer spread. When 100,000 cells were injected, there was an initial adhesion of cells to the endothelium of pulmonary vessels, and then a transient canceraemia. A steady extravascular migration of cancer cells was noted. After injection of 1,000,000 cells, there was a biphasic canceraemia. Theories have been advanced to account for this phenomenon.

Lung metastases rapidly grew to a large size, whilst hepatic metastases remained microscopic. The perivascular nature of the majority of metastases has been demonstrated. Very few small deposits were found in the bone marrow and none in the spleen.

We are most grateful to Dr. Warren H. Cole of the Research and Educational Hospital, Chicago, (where one of us—J.D.G.—held an Eli Lilli Travelling Fellowship) for his encouragement and help in the experimental work described in this paper ; also to Mr. Everett Hoppe for his technical help.

### REFERENCES

AMBRUS, J. L., AMBRUS, C. M., BYRON, J. W., GOLDBERG, M. E. AND HARRISON, J. W. E. —(1956) *Ann. N.Y. Acad. Sci.*, **63**, 938.

BASERGA, R. AND SAFFIOTTI, U.—(1955) *Arch. Path.*, **59**, 26.

CLIFFTON, E. E. AND GROSSI, C. E.—(1956) *Cancer*, **9**, 1147.

COMAN, D. R.—(1953) *Cancer Res.*, **13**, 397.

*Idem*, DELONG, R. P. AND MCCUTCHEON, M.—(1951) *Ibid.*, **11**, 648.

FISHER, E. R. AND FISHER, B.—(1959) *Cancer*, **12**, 926.

GRIFFITHS, J. D.—(1960) *Ann. R. Coll. Surg. Engl.*, **27**, 14.—(1961) 'British Surgical Practice. Surgical Progress '. London (Butterworths).

HAUSCHKA, T. S. AND LEVAN, A.—(1958) *J. nat. Cancer Inst.*, **21**, 77.

HEWITT, H. B. AND WILSON, C. W.—(1959) *Brit. J. Cancer*, **13**, 69.

IWASAKI, T.—(1915) *J. Path. Bact.*, **20**, 85.

JONASSON, O.—(1959) *Surg. Forum*, **9**, 577.

JONESCU, P.—(1931) *Z. Krebsforsch.*, **33**, 264.

LAWRENCE, E. A., BOWMAN, D. E., MOORE, D. B. AND BERNSTEIN, G. E.—(1952) *Surg. Forum*, **2**, 694.

LEVIN, I. AND SITTENFIELD, M. J.—(1911) *J. exp. Med.*, **14**, 148.

LONG, L., ROBERTS. S., MCGRATH, R. AND MCGREW, E.—(1959) *J. Amer. med. Ass.*, **170**, 1785.

LUCKE, B., BREEDIS, C., WOO, Z. P., BERWICK, L. AND NOWELL, P.—(1952) *Cancer Res.*, **12**, 734.

OVERSTREET, R. J. AND McDONALD, G. O.—(1958) *Surg. Forum.*, **8**, 161.
PAGET, S.—(1889) *Lancet*, i, 571.
PRINZMETAL, M., ORNITZ, E. M. JR., SIMKIN, B. AND BERGMAN, H. C—(1948) *Amer. J. Physiol.*, **152**, 48.
ROMSDAHL, M., CHU, E., HUME, R. AND SMITH, R.—(1961) *J. nat. Cancer Inst.*, **26**, 19.
SCHMIDT, M. B.—(1903) 'Die Verbreitungswege der Karzinome und die Beziehung generalisierter Sarkome zu den leukämischen Neubildungen'. Jena, (Gustav Fischer).
SNELL, G. D.—(1953) *J. nat. Cancer Inst.*, **13**, 1511.
TAKAHASHI, M.—(1915) *J. Path. Bact.*, **20**, 1.
TOBIN, C. E. AND ZARIQUIEY, M. O.—(1950) *Proc. Soc. exp. Biol., N.Y.*, **75**, 827.
WARREN, S. AND GATES, O.—(1936) *Amer. J. Cancer*, **27**, 485.
WATANAHE, S.—(1954) *Cancer*, **7**, 215.
WOOD, S. JR., HOLYOKE, E. D. AND YARDLEY, J. H.—(1961) *Canad. Cancer Conf.*, **4**, 167.
YOSHIDA, T.—(1955) *Ann. N.Y. Acad. Sci.*, **63**, 852.
ZEIDMAN, I.—(1957) *Cancer Res.*, **17**, 157.
ZEIDMAN, I., McCUTCHEON, M. AND COMAN, D. R.—(1950) *Ibid.*, **10**, 357.